# Mobile App Use for Insomnia Self-Management: Pilot Findings on Sleep Outcomes in Veterans

**DOI:** 10.2196/12408

**Published:** 2019-07-24

**Authors:** Erin D Reilly, Stephanie A Robinson, Beth Ann Petrakis, Eric Kuhn, Wilfred R Pigeon, Renda Soylemez Wiener, D Keith McInnes, Karen S Quigley

**Affiliations:** 1 Center for Social and Community Reintegration Research Edith Nourse Rogers Memorial VA Hospital Bedford, MA United States; 2 Center for Healthcare Outcomes and Implementation Research Edith Nourse Rogers Memorial VA Hospital Bedford, MA United States; 3 National Center for PTSD Veterans Affairs Palo Alto Health Care System Palo Alto, CA United States; 4 Stanford University School of Medicine Standford, CA United States; 5 Center of Excellence for Suicide Prevention Canandaigua VA Medical Center Canandaigua, NY United States; 6 University of Rochester Medical Center Rochester, NY United States; 7 Boston University School of Medicine Boston, MA United States; 8 Boston University School of Public Health Boston, MA United States; 9 Northeastern University Boston, MA United States

**Keywords:** cognitive behavioral therapy, mobile apps, insomnia, sleep apnea

## Abstract

**Background:**

Sleep disturbance is a major health concern among US veterans who have served since 2001 in a combat theater in Iraq or Afghanistan. We report subjective and objective sleep results from a pilot trial assessing self-management–guided use of a mobile app (*CBT-i Coach*, which is based on cognitive behavioral therapy for insomnia) as an intervention for insomnia in military veterans.

**Objective:**

The primary aim of this study was to evaluate changes in subjective and objective sleep outcomes from pre to postintervention.

**Methods:**

Subjective outcomes included the Insomnia Severity Index, the Pittsburgh Sleep Quality Inventory, and sleep-related functional status. A wearable sleep monitor (WatchPAT) measured objective sleep outcomes, including sleep efficiency, percent rapid eye movement (REM) during sleep, sleep time, and sleep apnea. A total of 38 participants were enrolled in the study, with 18 participants being withdrawn per the protocol because of moderate or severe sleep apnea and 9 others who dropped out or withdrew. Thus, 11 participants completed the full 6-week *CBT-i Coach* self-management intervention (ie, completers).

**Results:**

Completer results indicated significant changes in subjective sleep measures, including reduced reports of insomnia (*Z*=–2.68, *P*=.007) from pre (mean 16.63, SD 5.55) to postintervention (mean 12.82, SD 3.74), improved sleep quality (*Z*=–2.37, *P*=.02) from pre (mean 12.82, SD 4.60) to postintervention (mean 10.73, SD 3.32), and sleep-related functioning (*Z*=2.675, *P*=.007) from pre (mean 13.86, SD 3.69) to postintervention (mean 15.379, SD 2.94). Among the objective measures, unexpectedly, objective sleep time significantly decreased from pre to postintervention (*χ*^2^_2_=7.8, *P*=.02). There were no significant changes in percent REM sleep or sleep efficiency.

**Conclusions:**

These findings suggest that the *CBT-i Coach* app can improve subjective sleep and that incorporating objective sleep measures into future, larger clinical trials or clinical practice may yield important information, particularly by detecting previously undetected sleep apnea.

**Trial Registration:**

ClinicalTrials.gov NCT02392000; http://clinicaltrials.gov/ct2/show/NCT02392000

## Introduction

### Chronic Insomnia in Veterans

Sleep disturbance, especially chronic insomnia (difficulty falling and staying asleep), is a serious and prevalent problem among US veterans who have served since 2001 in a combat theater in Iraq and/or Afghanistan [1,2-5]. From 2000 to 2010, there was a 7-fold increase in the diagnosis of insomnia across veterans of all ages who were seeking care in the Veterans Health Administration (VHA) [5], with an upward trend in rates of insomnia and sleep apnea in military veterans continuing into this decade [6]. Sleep difficulties rarely occur in isolation from other physical and psychiatric concerns. For instance, insomnia and other self-reported sleep disturbances commonly cooccur with mental health diagnoses, such as posttraumatic stress disorder (PTSD) and depression [1,7-9], and they also cooccur with significant pain symptoms and other functional impairments [10-14]. Among veterans with multiple comorbidities, such as pain, PTSD, and traumatic brain injury, sleep disturbance rates are even higher. In VHA polytrauma clinics, 94% of US veterans who have served since 2001 in Iraq and/or Afghanistan report some sleep disturbance [4,15,16]. Thus, insomnia appears to be a prevalent problem for the most recent cohort of military veterans.

### Treating Chronic Insomnia With Cognitive Behavioral Therapy

Cognitive behavioral therapy for insomnia (CBTI) is a manualized therapy that uses cognitive behavioral techniques, including reconditioning, sleep restriction, sleep hygiene education, and relaxation skills, to help individuals manage and reduce chronic insomnia. There is strong evidence for the efficacy of CBTI [17], including brief interventions, such as 6 to 8 weeks of structured, weekly CBTI sessions [18,19], as well as briefer treatments [20]. Furthermore, CBTI has been successfully deployed in populations with comorbid health conditions [21], such as chronic pain [22-25], PTSD [17-19,26], and depression [27,28]. In addition, evidence from 2 uncontrolled studies of outpatient clinic patients [27] and a large randomized trial of internet-delivered CBTI [29] suggests that CBTI can reduce suicidal ideation, which is of paramount importance given the public health crisis of suicide among post-9/11 veterans [30]. Although the VHA has prioritized training additional clinicians to use CBTI, the demand for behavioral sleep treatments still outstrips supply [23]. In addition, adherence to CBTI can be poor, especially among younger, working-age veterans who face many competing life demands, including health concerns, work, and/or school and family needs [31,32]. Given these challenges, the use of mobile CBTI interventions has been increasing steadily over the last decade. Research suggests that telephone-delivered CBTI is feasible and acceptable [32], with many veterans preferring either individual-based or internet-delivered insomnia treatments rather than in-person, group treatment delivery [31]. This mirrors research in civilian populations as well, where electronically delivered versions of CBTI have been found to improve sleep in college students [33]. Thus, we hypothesized that use of a mobile app to deliver CBTI elements would be well received by veterans with chronic insomnia, given their treatment delivery preferences and high levels of interest in and use of computers and the internet for medical purposes [34]. A publicly available version of a mobile app to deliver CBTI elements, *CBT-i Coach*, was released in 2013 by the Veterans Affairs (VA) National PTSD Center and the Department of Defense National Center for Telehealth and Technology [35-37]. *CBT-i Coach* was based on a CBTI manual created by VA and university sleep researchers [38], and it includes sleep diaries, sleep health education, sleep restriction guidelines, and tools to encourage relaxation. Other features include reminders for bedtime/wake time, stopping caffeine, and scheduled wind down and worry time, with surveys to guide behavioral or environmental changes. For more details about the app, see Kuhn et al 2016 [36].

### Utilization of Subjective and Objective Sleep Measurement for Intervention Assessments

Subjective and objective measures of sleep often provide nonredundant data in the context of an intervention, as they often do not correlate highly in patients with insomnia [39], including some individuals with subjectively defined insomnia but objectively normal sleep [40]. Subjective/objective concordance varies as a function of health status, with lower concordance between subjective and objective sleep duration in those with poorer functional health, obesity, depressive symptoms, or lower sleep efficiency [39]. Objective and subjective sleep measures can differentially predict treatment outcomes [41]. As subjectively measured insomnia is related to numerous comorbid health outcomes (eg, chronic pain, posttraumatic stress, and depression), subjective measures are critical for measuring the impact of CBTI intervention success, and these are frequently measured through daily sleep diaries [42], self-report measures, such as the Pittsburgh Sleep Quality Inventory (PSQI) [43], or the Insomnia Severity Index (ISI) [44]). Given the difficulty younger veterans have with committing to the time required for in-person CBTI, further investigation of the impact of the *CBT-i Coach* app on subjective sleep in veterans is warranted. Measures of sleep disturbance, such as a reduced proportion of time spent in rapid eye movement (REM) sleep over a night and the occurrence of sleep apnea, have been proposed as potentially important objective sleep problems in veterans [3,45-48]. Information about REM abnormalities (eg, such as shorter more frequent bouts of REM [49-51]) also may be used as an indication of the need to assess for PTSD symptoms and/or to titrate PTSD treatments. The difficulty in efficiently capturing objective sleep data is a barrier to incorporating objective data into clinical practice. On the one hand, the costs and patient burden of laboratory-based overnight polysomnography are prohibitive. Furthermore, wrist-worn actigraphy devices measure sleep-wake activity, but they do not provide sleep stage information. As objective sleep measures in a self-management trial could have the benefit of improving the tailoring of CBTI interventions and sleep self-management, efforts to gather such data, using patient-friendly, well-validated devices, are worthwhile.

### This Study

This pilot study investigated the effects of a mobile insomnia self-management intervention. Self-management of insomnia was facilitated with the *CBT-i Coach* mobile app, including suggested activities in the app over a 6-week intervention period. Our primary aim was to assess the effectiveness of the *CBT-i Coach* app. We hypothesized there would be pre to postintervention improvements in subjective sleep outcomes (self-reported insomnia severity, sleep quality, and functional sleep) and objective sleep-related variables (total sleep time, sleep efficiency, and percent time in REM sleep). We also explored the relationship between demographic factors and self-reported mental and physical health outcomes, and we assessed the prevalence of positive sleep apnea screens in our sample.

## Methods

### Overview

This study was approved by the Institutional Review Board of the Edith Nourse Rogers Memorial veterans Hospital, Bedford, Massachusetts. We report subjective and objective sleep and functional health outcomes from a 6-week open trial pilot intervention of self-management–based use of the *CBT-i Coach* app. Owing to space restrictions, other measures of feasibility and usability testing of the features of this study will be reported elsewhere.

### Participant Recruitment

**Figure 1:**
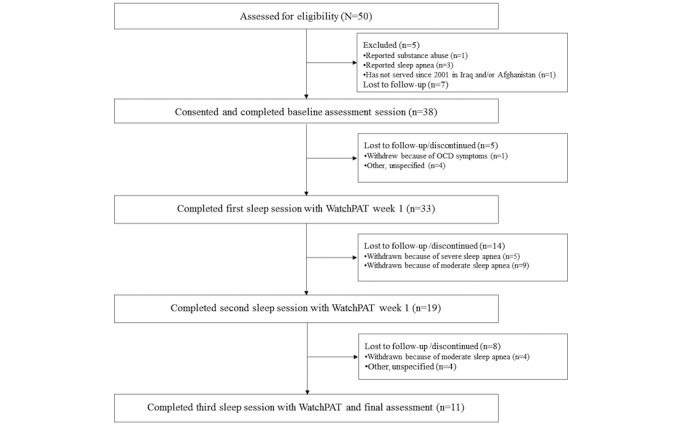
Consolidated Standards of Reporting Trials diagram of participant inclusion and attrition. OCD: obsessive-compulsive disorder.

Veterans were recruited via flyers, presentations, outreach to local community organizations, referrals from VA behavioral and sleep health providers, and recruitment letters. Recruitment letters were sent to a list of potentially eligible veterans that was generated by VA Informatics and Computing Infrastructure, followed by a telephone call no sooner than 2 weeks later to veterans who did not reply to the letter. Interested veterans were screened for study eligibility by phone. To be eligible, participants must have served since 2001 in a combat theater in Iraq and/or Afghanistan, reported current insomnia lasting at least 1 month, as defined by an ISI score greater than 10 [32,52], and impaired daytime functioning (as measured by endorsing *Much* or *Very Much* on ISI Item 7 about how much sleep problems interfere with daily functioning). Participants were excluded if they demonstrated moderate-to-severe cognitive impairment (defined by scores on the Telephone Mini Mental State Exam [53]), self-reported sleep apnea, periodic leg movements, or circadian rhythm disorder (delayed or advanced sleep phase). Figure 1 shows participant flow through the study.

### Participant Demographics

The mean age of the sample was 44.29 years, and the mean body mass index (BMI) was 29.0 kg/m^2^ (ie, in the overweight range). Participants (32 men, 6 women) had an average baseline ISI score of 15.42 (ie, moderate insomnia severity). Participants could identify with multiple racial categories, with 89% (34/38) participants identifying as white/Caucasian and 13% (5/38) participants identifying as Hispanic. A total of 37% (14/38) participants reported a combined average household income of US $50,000 to US $99,000 and another 37% (14/38) participants reported an average income over US $100,000. 94% (36/38) of participants reported that their health in general was good, very good, or excellent (94%). In addition, 55% (21/38) participants of the sample were married or living with a partner. The sample was well educated, with 61% (23/38) participants having an Associate’s or higher degree. See Table 1 for detailed demographic information.

**Table 1:** Descriptive statistics by enrollment status (N=38).

Characteristic		Withdrawn because of apnea (n=18)	Self-withdrew (n=9)	Completers (n=11)	Total (N=38)
Age (years), mean (SD)		42.22 (11.58)	44.11 (10.06)	47.82 (10.52)	44.29 (10.92)
BMI^a^, mean (SD)		30.68 (4.49)	27.77 (4.11)	27.22 (3.02)	29.00 (4.10)
Baseline ISI^b^, mean (SD)		15.33 (4.19)	14.11 (4.68)	16.63 (5.55)	15.42 (4.69)
**Race, n (%)**					
	White	15 (83)	9 (100)	10 (91)	34 (90)
	Black/African American	2 (11)	0	0	2 (5)
	Puerto Rican	2 (6)	0	1 (9)	2 (5)
	Filipino	0	1 (11)	1 (9)	2 (5)
	American Indian	0	0	1 (9)	1 (3)
	Other	2 (6)	0	1 (9)	2 (5)
**Ethnicity, n (%)**					
	Hispanic/Latino	3 (17)	1 (13)	1 (9)	5 (13)
	Not Hispanic/Latino	15 (83)	8 (89)	10 (91)	33 (86)
**Income US $, n (%)**					
	Less than 11,999	0	1 (11)	1 (9)	2 (5)
	12,000-24,999	3 (17)	0	0	4 (11)
	25,000-49,999	2 (11)	0	2 (18)	4 (11)
	50,000-99,000	6 (33)	4 (44)	4 (36)	14 (37)
	100,000+	7 (39)	3 (33)	4 (36)	14 (37)

^a^BMI: body mass index.

^b^ISI: Insomnia Severity Index.

### Description of the Intervention: Cognitive Behavioral Therapy-i Coach, Self-Management Guidance, and WatchPAT

The intervention comprised 6-week use of the *CBT-i Coach,* with added self-management support and a set of supplemental app-delivered worksheets to address behavioral concerns or prompt behavioral changes. All participants were loaned an Apple iPod Touch, on which the app was installed (iOS ver. 2.0) for the duration of the study.

#### Cognitive Behavioral Therapy-i Coach

*CBT-i Coach* offers sleep psychoeducation, tools for tracking sleep (eg, enables and can prompt for completion of daily sleep diaries and the ISI), and provides sleep hygiene recommendations, including cultivating a conducive sleep environment, engaging in regular exercise, and maintaining a healthy diet. Relaxation tools include multiple guided imagery audio clips, tips for winding down, breathing tools, and an audio-guided progressive muscle relaxation. A behavioral plan can also be reviewed and updated in *CBT-i Coach*, including setting reminders for when to go to sleep and get out of bed, complete sleep diaries and take ISI assessments, engage in scheduled worry time, and stop caffeine intake for the day. *CBT-i Coach* allows users to see graphs of their sleep diary data and ISI scores. We also created supplemental worksheets on the basis of the elements of the *Quiet Your Mind and* *Get to Sleep* manual [54]. These sleep worksheets were embedded in a separate app that participants could also access from the iPod Touch. Supplemental worksheets included Wakeful Activities, Coping Self-Statements, Constructive Worry, and a Relaxation Log.

#### Self-Management Guide

A self-management guide, in the form of a document accessible on the iPod Touch, along with a paper copy, provided week-by-week suggestions for using elements of the app and the worksheets. For each week of the 6-week intervention, the guide suggested what materials to read in the app, which features of the app to use, such as completing a daily sleep diary each morning, and which worksheets to complete.

#### WatchPAT Sleep Monitor

Objective sleep was recorded with a WatchPAT (model WP200U) sleep monitor (Itamar Medical Inc). The WatchPAT sleep monitor is a Food and Drug Administration–approved device that assesses objective sleep parameters, including a screen for obstructive sleep apnea. The WatchPAT is worn like a simple wristwatch with a plethysmographic-based finger-mounted probe and a small sensor on the chest to measure snoring. It is less obtrusive and less disruptive of sleep than either in-lab sleep assessments or in-home polysomnography. Moreover, the participant can use the device himself/herself, with simple instructions, which were provided via video on the iPod Touch and a laminated pamphlet.

### Measures

#### Demographics

Participants reported their age, gender, marital/partnered relationship status, race and ethnicity, and highest education level achieved. Height and weight were measured at the first visit.

#### Primary Outcomes

##### Subjective Sleep Measures

Self-reports of insomnia, sleep quality, and functional outcomes because of sleep were measured at baseline and final assessment visits using the ISI, the PSQI, and the Functional Outcomes of Sleep Questionnaire-10 item (FOSQ-10). The ISI has been shown to be sensitive to changes in insomnia severity with CBTI interventions [19,52,55], possible scores ranged from 0 to 28, with a higher score indicating more severe insomnia. The PSQI, a global measure of perceived sleep quality, has also been extensively validated and shown to be sensitive to change after CBTI [19,32]. Scores on the PSQI can range from 0 to 21, with a higher score indicating worse sleep quality. In the current sample, Cronbach alphas were acceptable (ISI=.83; PSQI=.75). The FOSQ-10 [56], a brief version of the original 30-item FOSQ [57], was used to assess the impact of sleepiness on functioning in everyday activities (Cronbach alpha=.89). Possible scores ranged from 5 to 20, with higher scores indicating better functional status.

##### Objective Sleep Measures

Objective sleep was measured via the WatchPAT and included total sleep time, total and percent time spent in light, deep, and REM sleep stages, apnea-hypopnea index (AHI), respiratory disturbance index (RDI), and number of awakenings. The WatchPAT is an FDA-approved portable diagnostic device that assesses sleep stages and detects probable sleep apnea with well-established validity in comparison to polysomnography-based measures of sleep apnea (eg, AHI and RDI; [58-65]). The WatchPAT calculates the proportion of REM sleep using a genetic algorithm (ie, a machine learning technique) to determine REM sleep onset and offset. The WatchPAT’s REM stage determination has also been validated against traditional polysomnography [66], as have its estimates of the duration of episodes of light versus deep sleep [67]. Participants were asked to wear the WatchPAT on their nondominant hand. Given the well-known “first night effect” in which sleep can be negatively impacted by sleep monitoring [68], especially in those with insomnia [69], participants recorded 2 nights of sleep at the beginning of the study, followed by 1 additional night at the end of the intervention, totaling 3 nights of objective sleep data.

#### Secondary Outcomes

Self-reported mental health and pain outcomes were measured at pre, mid, and postintervention visits. Mental health measures included assessments of nonspecific physical symptoms with the Patient Health Questionnaire-15 (PHQ-15) [70], depressive symptoms with the PHQ-9 [71], and PTSD symptoms with the PTSD Checklist (PCL-5) [63,72,73]. Each of these measures demonstrated good internal consistency (PHQ-15 alpha=.85, PHQ-9 alpha=.89, and PCL-5 alpha=.96). Pain severity was assessed using a 3-item subscale from the West Haven-Yale Multidimensional Pain Inventory (WHYMPI) [74], and pain-related functional health was measured using the Pain Disability Index (PDI) [75,76]. Both the WHYMPI and PDI demonstrated good internal consistency, with Cronbach alphas of .92 and .94, respectively.

### Procedure

At the first visit, participants completed the self-report questionnaires. They were given a printed version of the self-management guide, they were shown how to use it, and they were shown where to find an electronic copy on the iPod Touch, which they were given to use during the study. The researcher demonstrated use of the iPod Touch, *CBT-i Coach*, and the worksheet app and answered any questions. Participants were guided through setting a reminder to complete a weekly ISI assessment, and then they were guided through completing the first sleep diary and ISI on the *CBT-i Coach*. Participants were shown the WatchPAT, and they viewed a 4-min video on the iPod Touch that demonstrated how to set up and use the WatchPAT, including where and how to attach the sensors. The WatchPAT pamphlet also contained instructions and technical support information. Participants were instructed to complete daily sleep diaries and the activities in the self-management guide and to return with both devices for the second visit within the next week. During the second visit, the researcher downloaded the data from the WatchPAT and printed the associated sleep report. Those with an AHI score greater than 15 (indicative of probable moderate-to-severe sleep apnea) were excluded from further participation, and results were provided to their primary care provider for further assessment and clinical management. The report, which included the AHI score, total sleep times, and percentage of time in REM sleep, was reviewed with the participant by the researcher. Participants who were not excluded were asked to complete another night of WatchPAT monitoring within the coming week and then mail the device back to the investigators using a prepaid stamped envelope. The AHI score was again used to determine whether significant sleep apnea was likely (ie, a positive screen at the moderate or greater range), and, if so, the participant was withdrawn from the intervention and referred for further testing. At the midpoint of the intervention (about week 3-4), participants completed questionnaires with a researcher by phone and were scheduled for their postintervention visit (about week 6-7) and mailed a Watch PAT device. At the third and final in-person visit (ie, postintervention), participants returned both devices, completed final self-report questionnaires and a qualitative interview, and received their final WatchPAT report. WatchPAT reports were also shared with the participant’s primary care provider. Participants received US $15 each for the baseline, first WatchPAT, and midpoint visits, and they received US $40 at the completion of the postintervention visit, for a possible total of US $70 for completion of the entire study. Use of the *CBT-i Coach* app, specifically input of nightly sleep diaries, was high, with 9 of the 11 participants using the sleep diary portion of the app 85% of the time.

### Data Analysis

To address our primary aim, we evaluated whether subjective sleep outcome variables (ISI, PSQI, and FOSQ) changed from pre to postintervention, using Wilcoxon signed rank tests (nonparametric paired 2-tailed *t* tests). In addition, using repeated measures analysis of variance, we evaluated whether objective sleep outcome variables recorded from the WatchPAT—sleep time, REM percent of total sleep time, and sleep efficiency (ie, percent of total time in bed spent asleep vs awake)—changed across the 3 nights (2 nights early in the intervention and 1 at intervention end). Furthermore, using bivariate correlations, we explored the relationship among mental health symptom measures (ie, PHQ-15, PHQ-9, and PCL-5), pain severity (subscale from the WHYMPI), and subjective sleep outcomes for the entire enrolled sample (N=38). Finally, we used a chi-square test of independence to conduct exploratory analyses of sleep apnea status with demographic and mental health–related correlates of sleep apnea severity (ie, AHI in the mild, moderate, or severe range).

## Results

### Subjective Sleep Outcomes

Wilcoxon signed rank tests were used to compare the average scores for subjective sleep measures between pre- and posttest. Participants (n=11) showed a significant decrease in ISI scores (*Z*=–2.68, *P*=.007) from pre (median=15.00) to postintervention (median=13.00); a total of 9 participants noticed a reduction in reported insomnia severity over the 6-week intervention, and 2 participants reported no changes. Similarly, there was a significant decrease in PSQI scores (*Z*=–2.37, *P*=.02) from pre (median=12.50) to postintervention (median=12.00); a total of 8 participants noticed an improvement in sleep quality, 2 participants noticed their sleep being worse, and 1 participant reported no difference. Finally, there was a significant increase in FOSQ scores (*Z*=2.675, *P*=.007) from pre (median=14.33) to postintervention (median=16.33), with 9 participants reporting better functional outcomes at the end of the intervention that they attributed to better sleep, and 2 participants reporting no changes in functioning (see Table 2 for group descriptive statistics).

**Table 2:** Primary subjective sleep outcome measures at pre-, mid-, and postintervention.

Outcome	Preintervention		Midintervention		Postintervention	
	Mean (SD)	Median	Mean (SD)	Median	Mean (SD)	Median
ISI^a^	16.63 (5.55)	15.00	12.17 (4.39)	12.50	12.82 (3.74)	13.00
PSQI^b^	12.82 (4.60)	12.50	10.50 (2.65)	9.00	10.73 (3.32)	12.00
FOSQ^c^	13.86 (3.69)	14.33	15.26 (3.61)	15.25	15.379 (2.94)	16.33

^a^ISI: Insomnia Severity Index.

^b^PSQI: Pittsburgh Sleep Quality Index.

^c^FOSQ: Functional Outcomes of Sleep Questionnaire.

### Objective Sleep Outcomes

Friedman tests (nonparametric repeated measures) were also used to compare total sleep time recorded across 3 nights of objective sleep measurement provided by the WatchPAT, which revealed a statistically significant difference in total sleep time across the 3 monitored nights (*χ*^2^_2,11_=7.8; *P*=.02). Post hoc tests with Bonferroni correction revealed that there was a significant decrease in total sleep time (*P*=.004) from the first sleep session (median*=* 6 hours 53 min) to the second sleep session (median=5 hours 52 min) and from the first sleep session (*P*=.02, median=6 hours 53 min) to the third sleep session (median=6 hours 35 min). There was no statistically significant difference from the second to the third sleep sessions. There was also no significant effect of sleep session for the other objective sleep measures, including the percent REM sleep (*χ*^2^_2,11_=2.5; *P*=.29) or sleep efficiency, (*χ*^2^_2,11_=0.1; *P*=.91; see Table 3).

**Table 3:** Primary outcomes of objective sleep measures at sleep sessions.

Outcome	First sleep session		Second sleep session		Third sleep session	
	Mean (SD)	Median	Mean (SD)	Median	Mean (SD)	Median
Sleep time, hours	6.93 (1.39)	6.53	5.65 (1.45)	5.52	6.03 (1.71)	6.35
REM^a^ (%)	23.36 (7.67)	22.5	26.72 (6.94)	27.9	23.62 (7.06)	26.9
Sleep efficiency (%)	85.65 (6.02)	87.24	85.93 (6.69)	86.62	86.15 (8.43)	87.12

^a^REM: rapid eye movement.

### Relationship of Sleep to Mental Health and Pain Outcomes

Table 4 shows the bivariate correlations among mental health measures, pain severity, and subjective sleep outcomes, and Table 5 details descriptive statistics from baseline, midpoint assessment, and postintervention.

Higher scores on the ISI (ie, greater insomnia symptoms) were significantly and positively correlated with higher scores on the PHQ-15 (ie, greater nonspecific physical symptoms), PHQ-9 (ie, greater depressive symptoms), PCL-5 (ie, greater posttraumatic stress symptoms), and WHYMPI subscale (ie, greater pain severity). Higher scores on the FOSQ (ie, better functioning attributed to sleep) were significantly negatively correlated with nonspecific physical symptoms, depressive symptoms, posttraumatic symptoms, and pain severity (PHQ-15, PHQ-9, PCL-5, and WHYMPI subscale, respectively). In addition, higher scores on the PSQI (ie, poorer sleep quality) were significantly positively correlated with higher scores on the PHQ-15 (ie, greater nonspecific physical symptoms), PHQ-9 (ie, depressive symptoms), PCL-5 (ie, posttraumatic stress symptoms), and WHYMPI subscale (ie, pain severity). Finally, there were significant positive correlations between scores on the ISI and PSQI, and there were negative correlations of both measures with the FOSQ. Nonparametric Wilcoxon signed rank tests were run to assess changes between pre- and midpoint of the intervention (n=11) for the PHQ-9 (*P*=.23), PHQ-15 (*P*>.99), WHYMPI subscale (*P*=.55), and PDI (*P*=.28), as well as pre- and postintervention for the PCL-5 (*P*=.45), PHQ-9 (*P*=.12), PHQ-15 (*P*=.67), WHYMPI subscale (*P*=.53), and PDI (*P*=.18), with all changes nonsignificant.

**Table 4:** Correlation between subjective sleep and mental health measures (N=38).

Outcome	1	2	3	4	5	6	7
ISI^a^	—^b^	—	—	—	—	—	—
FOSQ^c^	–0.60^d^	—	—	—	—	—	—
PSQI^e^	0.55^d^	–0.39^f^	—	—	—	—	—
PCL-5^g^	0.55^d^	–0.69^d^	0.47^d^	—	—	—	—
WHYMPI^h^	0.41^f^	–0.36^f^	0.38^f^	0.49^d^	—	—	—
PHQ-15^i^	0.55^d^	–0.51^d^	0.49^d^	0.63^d^	.64^d^	—	—
PHQ-9^j^	0.48^d^	–0.72^d^	0.43^d^	0.79^d^	.33^f^	0.55^d^	—
Mean (SD)	15.42 (4.69)	13.9 (3.31)	12.05 (4.24)	22.45 (17.11)	5.53 (4.93)	8.61 (5.13)	8.82 (5.79)
Range	7-26	7-20	3-19	3-61	0-14	2-19	1-27

^a^ISI: Insomnia Severity Index.

^b^Correlation not applicable or redundant.

^c^FOSQ: Functional Outcomes of Sleep Questionnaire.

^d^*P*<.01.

^e^PSQI: Pittsburgh Sleep Quality Index.

^f^*P*<.05.

^g^PCL-5: Posttraumatic Stress Disorder checklist for Diagnostic and Statistical Manual of Mental Disorders-5.

^h^WHYMPI: West Haven-Yale Multidimensional Pain Inventory.

^i^PHQ-15: Patient Health Questionnaire-15.

^j^PHQ-9: Patient Health Questionnaire-9.

**Table 5:** Secondary outcome measures at pre-, mid-, and postintervention for completers (n=11).

Outcome	Preintervention		Midintervention		Postintervention	
	Mean (SD)	Median	Mean (SD)	Median	Mean (SD)	Median
PCL-5^a^	18.45 (16.03)	14.00	—^b^	—^b^	15.18 (11.50)	11.00
PHQ-9^c^	7.64 (3.98)	7.00	6.58 (4.96)	4.00	6.18 (3.79)	6.00
PHQ-15^d^	7.91 (5.43)	6.00	7.33 (5.40)	5.50	7.64 (4.61)	6.00
WHYMPI^e^	6.73 (4.54)	7.00	6.92 (5.30)	9.00	6.36 (4.48)	6.00
PDI^f^	22.18 (17.93)	21.00	16.33 (15.55)	11.50	15.45 (13.56)	15.00

^a^PCL-5: PTSD Checklist for DSM-5 (not administered midintervention).

^b^Measure not collected midintervention.

^c^PHQ-9: Patient Health Questionnaire-9.

^d^PHQ-15: Patient Health Questionnaire-15.

^e^WHYMPI: West Haven-Yale Multidimensional Pain Inventory.

^f^PDI: Pain Disability Index.

### Sleep Apnea Rates and Correlates

First or second night WatchPAT data revealed that 54.6% (18/33) of participants had possible moderate-to-severe apnea (ie, AHI score>15). These participants were withdrawn from further participation and were provided with a referral for further apnea testing. Of these participants, 89% (16/18) were male, 50% (9/18) had a BMI in the overweight (BMI=25-29 kg/m^2^, per the World Health Organization) and 39% (7/18) had a BMI in the obese (BMI>30 kg/m^2^) range. The mean age was 42.22 years (SD 11.58). More detailed demographic comparisons by enrollment status can be seen in Table 1. There were no significant relationships between having moderate-to-severe apnea and gender, *χ*^2^_2_=1.3, *P*=.25; BMI category, *χ*^2^_2_=3.9, *P*=.14; or age category, *χ*^2^_2_=0.2, *P*=.89; although greater BMI was significantly positively correlated with having a higher first-night AHI score, *r*=.39, *P*=.02. The number of female participants was small, likely meaning that the gender comparison was underpowered. Chi-square tests revealed that those with moderate or severe apnea were not more likely to have depressive symptoms, *χ*^2^_1_=0.7, *P*=.68, or posttraumatic symptoms, *χ*^2^_1_ =1.0, *P*=.31, than those with no or mild apnea. There was a marginal difference in nonspecific physical symptoms, such that those with moderate or severe apnea reported marginally more nonspecific physical symptoms, *χ*^2^_1_=3.3, *P*=.07. Finally, a chi-square test of independence was performed to examine the relationship between moderate-to-severe apnea and pain measures, which revealed no significant relationships—pain severity, *χ*^2^_1_=1.8, *P*=.67; PDI, *χ*^2^_1_=0.9, *P*=.81; and sleep-related functioning via the FOSQ, *χ*^2^_1_=0.5, *P*=.46.

## Discussion

### Principal Findings

We conducted a study of a CBTI mobile app (*CBT-i Coach*), with supplemental self-management guided use of worksheets in veterans reporting chronic insomnia and assessed changes in subjective and objective sleep measures. Veterans who completed the 6-week mobile sleep intervention reported improvements in insomnia severity, sleep quality, and sleep-related functional outcomes. No changes in objective sleep measures were observed, except that time asleep decreased, even though subjective sleep was rated as better. Improvements in subjective sleep were found on 3 common subjective sleep assessments, the ISI, PSQI, and the FOSQ. In the context of clinical guidelines, the ISI decreased from a score indicative of moderate clinical insomnia severity at pretest (≥15) to a score below the subthreshold level of insomnia at posttest (scores 8-14) [52]. A score on the PSQI of 5 or greater has been suggested as indicative of poor sleep quality, and it often prompts recommendation of further follow-up with a health care provider [43]. The PSQI did not decrease below the suggested cutoff score of 5, although it did significantly decrease. In addition, functional sleep outcomes, as measured by the FOSQ, significantly improved as well. The findings of this small pilot study support the possibility that veterans can use these technology-based tools to self-manage their chronic insomnia and experience improved sleep quality and functioning, with minimal clinical resources. Although subjective sleep measures improved in completers, objective sleep measures did not significantly improve at the end of the intervention. In fact, contrary to our hypothesis, total sleep time decreased significantly from the first night to subsequent nights (both early in the intervention and postintervention). This decrease in sleep time may be related to when the participants chose to wear the WatchPAT. Individuals typically completed their first night’s sleep on the same day as their first lab visit or on a weekend to allow more time for sleep, suggesting the possibility that they completed their first night of sleep monitoring on a day when sleep time could be more easily extended; thus, it was perhaps atypical of their usual daily sleep schedule. Interestingly, there was no difference from the second to the third sleep sessions in total sleep time. It is possible that the first night was unusual for participants. As it was their first assessment with the WatchPAT, patients may have made more of a concerted effort during the first sleep session to sleep longer, to obtain a valid sleep assessment. Future research would benefit from several objective assessments to get a more accurate assessment of sleep time and possible first-night effects when using at-home sleep monitors.

### Identifying Sleep Apnea Through At-Home Wearable Devices

The WatchPAT findings also revealed a relatively high prevalence of sleep apnea in this largely middle-aged, male sample of veterans. Moderate-to-severe sleep apnea was not predicted by psychological or health-related factors, except for higher BMIs being associated with high apnea scores, as assessed by AHI scores, consistent with previous studies [77-79]. More than 54% of the sample that completed at least one WatchPAT measurement showed probable moderate-to-severe sleep apnea. This was unexpectedly high as compared with epidemiologically based community samples indicating approximately 5% rates in the general population [80].

Our rate is high even when compared with other veteran samples, including a veteran epidemiological sample of those seeking health care in the VA, with a sleep apnea diagnosis rate of 4.5% [5] or an intervention sample where 38.5% of the sample screened positive for self-reported sleep apnea symptoms [19]. Apnea rates appear to have increased as prior veteran administrative data sources revealed a prevalence of diagnosed sleep apnea of only about 3.5% [48,81]. Thus, our prevalence rate for sleep apnea is considerably higher than expected, although the sample is small and self-selected; thus, the rate may not be representative of the larger population. Increasing age of the current veteran population may partially explain this increase, although Alexander et al [5] report an age-adjusted increase in apnea rates of 3.7% from 2000 to 2010. The higher prevalence rate for this study may also be because of apnea detection using a well-validated home sleep monitoring device rather than using self-reported sleep apnea symptoms or self-reported diagnoses, which is likely to lead to an underestimation of apnea in the population. Overall, these findings suggest that use of an at-home, nonobtrusive, wearable sleep device to screen for sleep apnea is not only practical, efficient, and effective but it also could be used to identify otherwise occult, and therefore untreated, sleep apnea.

### Future Application of Mobile-App Supported Sleep Self-Management in the Veterans Affairs

The VA has undertaken a whole health approach to help providers focus on what matters most to their patients, and sleep is one of the main health behaviors included in these discussions. The VA is committed to the development of mobile apps to support patients with a broad range of health conditions. To further this goal, our findings suggest that use of a home sleep monitor in veterans with insomnia may also assist clinicians in detecting unrecognized cases of sleep apnea and, as a result, lead to better apnea detection and more appropriate treatment of sleep disturbance. Utilizing these technologies also may help veteran patients feel empowered to improve their sleep health, potentially become more engaged in managing their own health conditions and more invested in making positive health behavior changes. It will be useful for future studies to determine how behavioral and sleep health clinicians in the VA (and elsewhere) can better incorporate these technologies into their work with patients.

### Limitations and Future Directions

Limitations of this pilot study include the small sample of intervention completers, the lack of a control group, the night-to-night variability in objective sleep measurement, the lack of follow-up data to see if the improvements in subjective sleep lasted after the intervention ended, the potential influence of social desirability, and the multicomponent nature of the intervention. The participants who withdrew of their own accord or discontinued in the study cited difficulties with continuing, including family and employment factors. This may have led to self-selection biases in the completer sample, as those individuals who completed the intervention may have been more likely to use mobile apps for health-related concerns relative to those who dropped out of the study. Among those participants who were not excluded because of sleep apnea (N=15), 73% (11/15) participants completed the intervention. Future research on the use of mobile sleep apps and at-home sleep monitors will need to consider participant factors that may lead to attrition. The small completer sample size may have impacted the effect sizes of the subjective and objective sleep findings; small sample sizes can drastically affect the ability to detect statistical power and reduce the likelihood that a statistically significant difference reflects a reproducible difference by overestimating effect size [82]. Consequently, the results here must be replicated in a larger sample before strong inferences can be made. Finally, future studies will need to utilize a control group to ensure that changes are because of the intervention effects and not because of potential demand characteristics or other nonspecific changes with the passage of time. We are currently conducting a randomized controlled 2-arm (experimental and control arms) study. Another limitation of this study includes the use of self-reported outcomes, which could have been influenced by social desirability. However, these well-validated measures were self-administered, which has been documented to reduce the effect of social desirability [83]. Finally, without a multiarm randomized controlled trial, the combined use of the *CBT-i Coach* app, self-management guide, and WatchPAT makes it impossible to attribute any changes in sleep measurements to the *CBT-i Coach* app itself. Other technologies may have emphasized the importance of paying attention to sleep patterns. Such feedback and symptom monitoring are critical components of CBTI, which likely enhanced the intervention [36].

### Conclusions

This study contributes to growing literature on the efficacy of cognitive behavioral therapy–based approaches in the treatment of insomnia, suggesting that less resource-intensive treatment modalities, such as self-management–based mobile apps, can provide clinically useful tools in the management of insomnia in some patients. Furthermore, these findings align well with the call to treat sleep disturbance in veterans and minimize work-related sleep disruptions in veteran active-duty military personnel, alongside greater acknowledgment of the importance of sleep by the military [84]. The prevalence of previously undetected sleep apnea uncovered by our approach to assessment was striking, and it merits further investigation. Integrating mobile sleep apps and wearable devices constitutes a promising area for helping veterans manage their health needs, and it merits future research to inform best practices for integrating these self-management options into clinical care throughout the VA.

## Abbreviations

AHI: apnea-hypopnea index
BMI: body mass index
CBTI: cognitive behavioral therapy for insomnia
FOSQ-10: Functional Outcomes of Sleep Questionnaire-10 item
ISI: Insomnia Severity Index
PCL-5: PTSD Checklist
PDI: Pain Disability Index
PHQ-9: Patient Health Questionnaire-9
PHQ-15: Patient Health Questionnaire-15
PSQI: Pittsburgh Sleep Quality Inventory
PTSD: posttraumatic stress disorder
RDI: respiratory disturbance index
REM: rapid eye movement
VA: Veterans Affairs
VHA: Veterans Health Administration
WHYMPI: West Haven-Yale Multidimensional Pain Inventory
